# Mitochondrial DNMT3A and DNA methylation in skeletal muscle and CNS of transgenic mouse models of ALS

**DOI:** 10.3389/fncel.2013.00279

**Published:** 2013-12-25

**Authors:** Margaret Wong, Barry Gertz, Barry A. Chestnut, Lee J. Martin

**Affiliations:** ^1^Department of Pathology, Division of Neuropathology, Johns Hopkins University School of MedicineBaltimore, MD, USA; ^2^Department of Pathology, Pathobiology Graduate Program, Johns Hopkins University School of MedicineBaltimore, MD, USA; ^3^Department of Neuroscience, Johns Hopkins University School of MedicineBaltimore, MD, USA

**Keywords:** ALS, DNA pyrosequencing, Dnmt1, Dnmt3a, mitochondrial DNA, 5-methylcytosine, mitochondria, motor neuron

## Abstract

Cytosine methylation is an epigenetic modification of DNA catalyzed by DNA methyltransferases. Cytosine methylation of mitochondrial DNA (mtDNA) is believed to have relative underrepresentation; however, possible tissue and cell differences in mtDNA methylation and relationships to neurodegenerative disease have not been examined. We show by immunoblotting that DNA methyltransferase 3A (Dnmt3a) isoform is present in pure mitochondria of adult mouse CNS, skeletal muscle, and testes, and adult human cerebral cortex. Dnmt1 was not detected in adult mouse CNS or skeletal muscle mitochondria but appeared bound to the outer mitochondrial membrane. Immunofluorescence confirmed the mitochondrial localization of Dnmt3a and showed 5-methylcytosine (5mC) immunoreactivity in mitochondria of neurons and skeletal muscle myofibers. DNA pyrosequencing of two loci (D-loop and 16S rRNA gene) and twelve cytosine-phosphate-guanine (CpG) sites in mtDNA directly showed a tissue differential presence of 5mC. Because mitochondria have been implicated in the pathogenesis of amyotrophic lateral sclerosis (ALS), but the disease mechanisms are uncertain, we evaluated mitochondrial Dnmt3a and 5mC levels in human *superoxide dismutase-1* (SOD1) transgenic mouse models of ALS. Mitochondrial Dnmt3a protein levels were reduced significantly in skeletal muscle and spinal cord at presymptomatic or early disease. Immunofluorescence showed that 5mC immunoreactivity was present in mitochondria of neurons and skeletal myofibers, and 5mC immunoreactivity became aggregated in motor neurons of ALS mice. DNA pyrosequencing revealed significant abnormalities in 16S rRNA gene methylation in ALS mice. Immunofluorescence showed that 5mC immunoreactivity can be sequestered into autophagosomes and that mitophagy was increased and mitochondrial content was decreased in skeletal muscle in ALS mice. This study reveals a tissue-preferential mitochondrial localization of Dnmt3a and presence of cytosine methylation in mtDNA of nervous tissue and skeletal muscle and demonstrates that mtDNA methylation patterns and mitochondrial Dnmt3a levels are abnormal in skeletal muscle and spinal cord of presymptomatic ALS mice, and these abnormalities occur in parallel with loss of myofiber mitochondria.

## Introduction

Methylation of DNA on carbon five of cytosine (5-methylcytosine, 5mC) in cytosine-phosphate-guanine (CpG) dinucleotides is an epigenetic modification of DNA used by cells to regulate nuclear gene expression (Jones and Takai, 2001; Bird, 2002; Brenner and Fuks, 2006). Cytosine methylation signals through 5mC-binding proteins to remodel chromatin and downregulate gene expression. Cytosine methylation is mediated by a family of DNA methyltransferase (Dnmt) enzymes (Cheng, 1995). Dnmt1 is the most abundant isoform in proliferating cells and displays a preference for hemimethylated substrates and is targeted to replication forks, acting to maintain DNA methylation patterns during cell replication (Robertson, 2001), and to DNA repair sites (Mortusewicz et al., 2005). Recently, mutations in the *Dnmt1* gene have been identified as a cause of hereditary sensory and autonomic neuropathy type 1 (Klein et al., 2011) and autosomal dominant cerebellar ataxia, deafness, and narcolepsy (Winkelmann et al., 2012). Dnmt2 (also called tRNA methyltransferase-1) transfers methyl groups to RNA instead of DNA (Schaefer and Lyko, 2010; Motorin and Helm, 2011). DNA methyltransferase 3A (Dnmt3a) and Dnmt3b function as *de novo* methyltransferases because they methylate hemimethylated DNA and also completely unmethylated DNA (Okano et al., 1999; Xie et al., 1999). Dnmt3L functions as an essential regulatory cofactor for Dnmt3a (Jia et al., 2007). Nuclear DNA methylation attracts attention because it has been implicated in normal cell and tissue development and differentiation (Geiman and Muegge, 2010) and in human diseases, including cancer (Calvanese et al., 2009), Rett syndrome (Amir et al., 1999), and, more recently, neurodegenerative diseases (Chestnut et al., 2011; Klein et al., 2011; Winkelmann et al., 2012; Martin and Wong, 2013).

Studies of mitochondrial DNA (mtDNA) cytosine methylation and the Dnmts that regulate mtDNA methylation are not as common as studies of nuclear DNA methylation; moreover, many mitochondrial-based mechanisms of disease have been implicated in amyotrophic lateral sclerosis (ALS) (Beal, 2005; Martin, 2010, 2012; Reddy and Reddy, 2011; Panov et al., 2012; Santa-Cruz et al., 2012), but mtDNA methylation and mitochondrial Dnmts have not been studied in ALS. Earlier work tends to minimize the occurrence and importance of mtDNA cytosine methylation. Some studies of cultured cells have found mtDNA cytosine methylation in mouse fibroblastoid cells (Pollack et al., 1984) and human fibroblasts (Shmookler Reis and Goldstein, 1983). In the latter study, ~2–5% of CCGG sites were fully methylated. Other studies have found underrepresented or no mtDNA cytosine methylation (Maekawa et al., 2004). However, the earlier studies that failed to observe mtDNA cytosine methylation were done on mitochondria isolated from blood. More recently, it has been reported that Dnmt1 localizes to mitochondria in cultured mouse embryonic fibroblasts and human colon carcinoma cells (Shock et al., 2011) and that Dnmt3a localizes to mouse brain and spinal cord mitochondria (Chestnut et al., 2011). Studies have yet to examine comparatively mtDNA methylation in different adult tissue types *in vivo* to test the hypothesis that there is tissue specificity for mtDNA cytosine methylation. Moreover, the physiological significance and potential pathophysiological relevance of mtDNA methylation in ALS merit exploration in light of findings that Dnmt3 is localized to neuronal mitochondria and appears to be upregulated in human ALS neurons and mouse spinal motor neurons during their degeneration (Chestnut et al., 2011).

In the current study, we demonstrate that Dnmt3a and 5mC are in mitochondria of adult mouse and human tissue types that are mostly excitable tissues (nervous and muscle) and that tissue Dnmt3a levels are reduced and 5mC immunoreactivity accumulates in skeletal muscle myofibers and spinal cord motor neurons in mouse models of ALS. We also show directly by pyrosequencing that mtDNA from these tissues contains 5mC and that the levels of 5mC in the 16S rRNA gene are increased in transgenic mouse models of ALS.

## Materials and methods

### Mice

Wildtype and human *superoxide dismutase-1* (hSOD1) transgenic (tg) mice were used with approval from the institutional Animal Care and Use Committee. For studies of normal DNMT3a and 5mC localizations and distributions, non-tg adult C57BL/6 and SV129 mice (*n* = 20–25) were used at 2–6 months of age. Several different tg mouse lines were used. One line was hemizygous for a low copy number of hSOD1-G37R mutant allele driven by the endogenous human promoter (line 29) derived from a founder B6.Cg-Tg SOD1-G37R 29Dpr/J (stock # 008229, The Jackson Laboratory, Bar Harbor, MA). Another tg mouse line (hSOD1-wildtype) expressed normal wildtype human *SOD1* gene (B6SJL-TgN[SOD1] 2Gur, stock #002297, The Jackson Laboratory) at high levels (copy number ~25) driven by the endogenous human promoter. These two tg lines thus expressed hSOD1 ubiquitously in many tissues (Gurney et al., 1994; Martin et al., 2007, 2009; Gertz et al., 2012). We also used tg mice with skeletal muscle-restricted expression of hSOD1-G37R, -G93A, and -wildtype variants (designated as hSOD1^mus^) which have been described previously (Wong and Martin, 2010). All the lines of hSOD1 tg mice were studied at presymptomatic or early to middle stages of disease. Control mice were age-matched, non-tg littermates. Mouse genotypes were identified by PCR. Mitochondrial fractions were prepared from 5–10 mice/genotype.

### Preparation of crude mitochondrial fractions from skeletal muscle and heart

**Figure 1:**
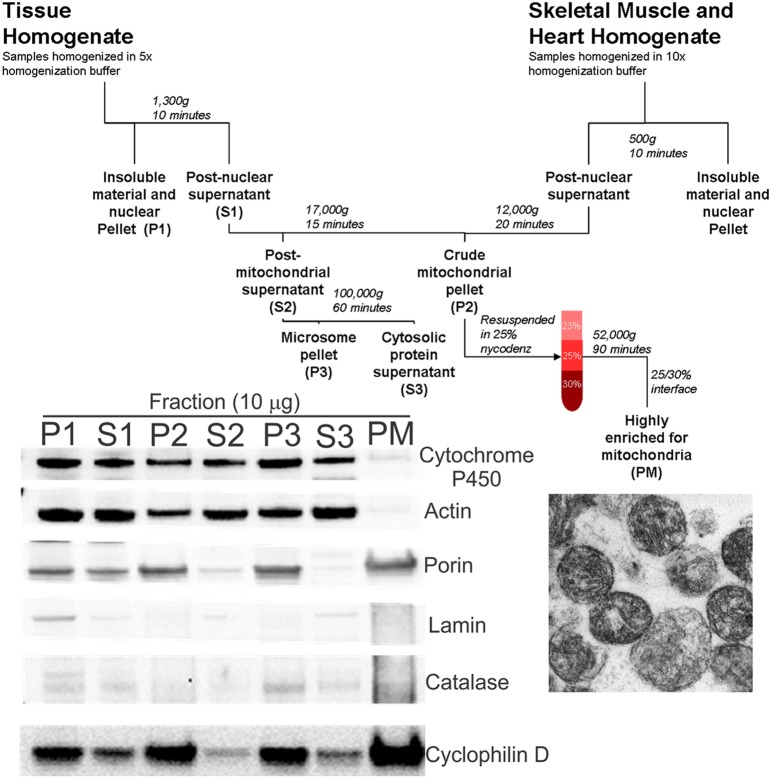
**Schematic diagram of tissue fractionation and experimental verification of mitochondrial enrichment and purity**. Tissues were homogenized in appropriate buffer using a PT3100 polytron. After centrifugation to remove tissue and cellular debris, a crude mitochondrial pellet was obtained (fraction P2). The pellet was resuspended in 25% nycodenz and a highly pure mitochondrial fraction (fraction PM) was obtained by discontinuous gradient centrifugation. Bottom left panel: Immunoblotting of the various fractions confirms the pure mitochondria (PM) fraction to be highly enriched for mitochondria (porin and CyPD), with very little or no contamination by cytoplasmic (actin), microsomal (cytochrome P450), nuclear (lamin), and peroxisomal/lysosomal (catalase) compartments. Bottom right panel: Electron microscopy on PM fractions confirmed visually the very high enrichment of mitochondria.

Tissue mitochondrial preparations were isolated from fresh tissues with no prior freezing or fixation. For fresh tissue harvesting, mice were deeply anesthetized and decapitated; organs and tissues were immediately removed, weighed, and placed in ice-cold phosphate-buffered saline (PBS). Muscle crude mitochondrial fractions were obtained using a modification (Figure 1) of a described method (Bhattacharya et al., 1991). The tissue was finely minced using a razor. The pieces were resuspended in 10 ml/g digestion buffer (100 mM sucrose, 10 mM EDTA, 100 mM Tris-HCl, 46 mM KCl, pH 7.4) with 0.1 mg/ml nagarse (Sigma-Aldrich) and were digested at room temperature for 10–20 min. The digest was placed in an equal volume of homogenization buffer consisting of digestion buffer with 5 mg/ml BSA and complete protease inhibitor (Roche Diagnostics). The tissue pieces were allowed to settle, the buffer was aspirated, and the tissue was resuspended in an equal volume of homogenization buffer. The tissue was homogenized using a PT 3100 Polytron (Kinematica) at 20,000 rpm on ice. The homogenate was centrifuged at 500 g for 10 min and the resulting supernatant was removed to a fresh tube. The supernatant was then centrifuged at 12,000 g for 20 min. The resulting pellet was the crude mitochondrial fraction.

### Preparation of crude mitochondrial fraction from tissues other than striated muscle

Crude mitochondrial fractions of organs were obtained using a described method (Okado-Matsumoto and Fridovich, 2001) with slight modifications (Figure 1). Brain, spinal cord, liver, kidney, colon, and spleen were homogenized in nycodenz homogenization buffer (210 mM mannitol, 70 mM sucrose, 10 mM Tris, 1 mM EDTA, and protease inhibitor, pH 7.5) at a 1:5 (w/v) ratio using a PT 3100 Polytron at 20,000 rpm on ice. The homogenate was centrifuged at 1300 g for 10 min and resultant supernatant removed to a fresh tube. The supernatant was then centrifuged at 17,000 g for 10 min. The resulting pellet was the crude mitochondrial fraction.

### Generation of pure mitochondrial fraction

**Figure 2:**
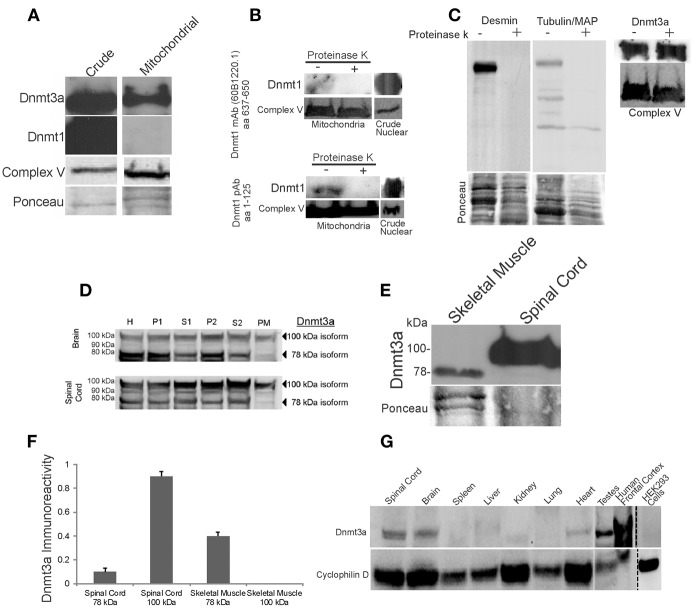
**Dnmt3a is present in skeletal muscle and CNS mitochondria of adult mouse and human**. **(A)** Western blots showing that Dnmt3a, but not Dnmt1, is present in adult mouse skeletal muscle mitochondria. Dnmt1 was present in crude tissue fractions but was undetectable in pure mitochondrial fractions even with prolonged overexposures as shown. Western blot for the mitochondrial marker complex V is used to verify mitochondrial enrichment of the fraction. Ponceau S-stained membrane is used to show protein loading. **(B)** Experiments that establish that Dnmt1 is bound to the outer surface of mitochondria. Freshly isolated intact skeletal muscle mitochondria were treated (+) with agarose-conjugated proteinase K to digest surface-bound proteins or were not treated with proteinase K (−). Mitochondria were lysed and proteins were fractionated by SDS-PAGE and western blotted for Dnmt1 using two different antibodies that detect different amino acid (aa) domains of the protein. Protein loading was revealed by reprobing the blots for complex V. Crude nuclear fractions were used as a positive control. **(C)** Experiments that establish that Dnmt3a is within mitochondria and not merely bound to the outer surface of mitochondria. Freshly isolated intact skeletal muscle mitochondria were treated with agarose-conjugated proteinase K to digest surface-bound proteins. The efficacy of digestion is shown by the loss of desmin and tubulin/MAP immunoreactivities in the treated samples, but Dnmt3a immunoreactivity was not attenuated by proteinase K digestion. Protein loading is shown by Ponceau S staining of membranes. Mitochondrial enrichment is shown by complex V immunoreactivity. **(D)** Adult mouse brain and spinal cord were homogenized, fractionated and immunoblotted with antibody to Dnmt3a. Lanes were loaded with equivalent amounts of protein from the crude homogenate (H) and different fractions, including nuclear-enriched and insoluble material (P1), post-nuclear supernatant (S1), crude mitochondria (P2), post-mitochondrial supernatant (S2), and pure mitochondria (PM). See Figure 1 for the validation of this fractionation method. The 100 kDa form of Dnmt3a was present in all fractions, including the pure mitochondria, while the 78 kDa form was present in all fractions except the mitochondria. **(E)** Western blot comparing the levels of Dnmt3a and isoform specificity in skeletal muscle (100 μg protein) and spinal cord (50 μg protein). Dnmt3a expression is greater in spinal cord compared to skeletal muscle. The 78 kDa isoform predominates in skeletal muscle while the 100 kDa isoform predominates in spinal cord. **(F)** Graph showing the relative levels of Dnmt3a isoform immunoreactivity determined by immunobloting of pure mitochondria isolated from adult mouse skeletal muscle and spinal cord. Values are mean ± SD. The 100 kDa isoform was not detected in skeletal muscle even after long exposures. **(G)** Immunoblot for Dnmt3a in pure mitochondria (PM) fractions from different types of adult mouse tissues, human brain, and a human cell line. The 100 kDa isoform of Dnmt3a was present in mouse spinal cord, brain, heart, testes, and human brain cerebral cortex. Dnmt3a immunoreactivity was low or undetectable in mouse spleen, liver, kidney, and lung and in human embryonic kidney cells. Western blot for the mitochondrial marker cyclophilin D is used to show protein loading.

A highly enriched mitochondrial fraction was obtained by nycodenz (Axis-Shield) discontinuous gradient centrifugation (Okado-Matsumoto and Fridovich, 2001). Gradients were prepared by diluting 50% nycodenz solution (5 mM Tris, 3 mM KCl, 0.3 mM EDTA, 25 g nycodenz, pH 7.5, prepared in 50 ml of double-distilled water) with the appropriate volume of dilution buffer (0.75 g NaCl, 5 mM Tris, 3 mM KCl, 0.3 mM EDTA, pH 7.5, prepared in 100 ml of double-distilled water) to obtain an iso-osmotic solution. The crude mitochondrial fractions were resuspended in 3 ml 25% nycodenz buffer, layered onto 2.5 ml 30% nycodenz buffer, and overlaid with 2.5 ml 23% nycodenz buffer. Samples were centrifuged at 52,000 g for 90 min in a swinging bucket rotor (Sorval TH641). The pure mitochondria (PM) fraction was isolated from the 25%/30% interface. This fraction was highly enriched in mitochondria (Figure 1, blot inset) as demonstrated by the absence of markers for nucleus (lamin), endoplasmic reticulum/microsomes (cytochrome P450 reductase), peroxisomes (catalase), and cytosol (actin), and the enrichment in mitochondrial markers, including porin (voltage-dependent anion channel, VDAC), cyclophilin D, and complex V (Figures 1, 2A–C,G). Aliquots of freshly prepared PM samples were fixed in 2% glutaraldehyde, embedded in plastic, and thin-sectioned for electron microscopy (EM) to further validate the mitochondrial purification using this approach (Figure 1).

To confirm that Dnmt protein was localized within mitochondria rather than being bound to the surface of mitochondria, proteinase K digestion of freshly isolated intact mitochondria was performed. PBS-resuspended mitochondria were incubated in 5 mg/ml agarose-conjugated proteinase K (Sigma) for 30 min at 37°C, and then centrifuged and washed in PBS for several cycles to remove proteinase K.

### Immunoblotting

Tissue subcellular fractions were separated by SDS-PAGE on 4–12% NuPage gels (Invitrogen) under denaturing and reducing conditions. Gels were transferred to a nitrocellulose membrane using the Xcell surelock system (Invitrogen). The membranes were stained with Ponceau S (Sigma) to determine transfer efficiency, destained, blocked with 1% BSA/0.05% Tween 20/TBS and incubated with primary antibodies: rabbit polyclonal anti-cytochrome P450 reductase (Stressgen) at 1:1000; mouse monoclonal anti-actin (Chemicon) at 1:1000; mouse monoclonal anti-porin (Mitosciences) at 1:3000; rabbit polyclonal anti-lamin (Millipore) at 1:500; sheep polyclonal anti-catalase (Biodesign International) at 1:2000; mouse anti-cyclophilin D (Mitosciences) at 1:3000; mouse monoclonal anti-complex V (Molecular Probes Invitrogen) at 1:10,000; rabbit polyclonal and mouse monoclonal anti-Dnmt3a (Cell Signaling, Abgent, and Alexis) at 1:100–1:500, rabbit polyclonal and mouse monoclonal anti-Dnmt1 (Bethyl, Novus, and Alexis) at 1:500–1:5000, and rabbit polyclonal antibody to Dnmt3b (Abcam) at 1:250. The details of the Dnmt antibodies used are shown in Table 1. To confirm the efficacy of proteinase K digestion of putative mitochondrial surface-tethered proteins, treated skeletal muscle mitochondria were probed with antibodies to tubulin/microtubule associated proteins (1:1000, Sigma) and desmin (1:5000, Sigma). Antibody to LC3A (Cell Signaling) was used as a mitochondrial autophagy marker. Blots were washed, incubated with secondary antibody (1:10,000–1:50,000), and developed using ECL (Pierce Supersignal West Pico). The membranes were imaged using a CCD camera and BioRad Quantity One software or were developed using X-ray film. Immunoreactivity was quantified using ImageJ.

**Table 1:** **Antibodies to Dnmt isoforms screened in this study**.

**Dnmt target**	**Antibody clonality**	**Immunogen**	**Company and product**	**Comment**
Dnmt1	Mouse monoclonal 60B1220.1	Synthetic peptide corresponding to aa 637–650 (central region)	Alexis (Enzo) ALX-804-369	Good for mouse skeletal muscle and CNS westerns
Dnmt1	Rabbit polyclonal	Synthetic peptide corresponding to aa 1–125 (N-terminus)	Novus Biologicals NB100-264	Good for mouse skeletal muscle and CNS westerns
Dnmt1	Rabbit polyclonal	Synthetic peptide corresponding to ~ aa 574–846 (encoded by exon 25)	Bethyl Laboratories BL961	Suitable for mouse tissue westerns
Dnmt3a	Mouse monoclonal 64B1446	Mouse recombinant Dnmt3a (C-terminal epitope aa 705–908)	Alexis (Enzo) ALX-804-370	Good for mouse skeletal muscle and CNS westerns and immunohistochemistry
Dnmt3a	Rabbit polyclonal	N-terminus of human Dnmt3a	Cell Signaling Technology 2160	Suitable for mouse skeletal muscle and CNS westerns and immunohistochemistry
Dnmt3a	Rabbit polyclonal	Synthetic peptide corresponding to aa 400–500 of human Dnmt3a	ABGENT AP1034a	Suitable for mouse skeletal muscle and CNS westerns
Dnmt3b	Mouse monoclonal 52A1018	Mouse recombinant Dnmt3b	Abcam Ab13604	No detection of Dnmt3b in skeletal muscle mitochondria

### Immunofluorescence

Age-matched non-tg and hSOD1^mus^-G37R, -G93A, and -wildtype tg mice (12–15 months of age) received an anesthetic overdose and were perfused transcardially with 4% paraformaldehyde. The group sizes were 3–4 mice per genotype. Spinal cord, skeletal muscle, and testes were removed and cryoprotected in 20% glycerol and then frozen and cut (40 μm) on a sliding microtome. Sections were permeabilized in 0.4–1% Triton-X100/PBS, blocked in 10% normal donkey serum or goat serum/0.1% Triton/PBS, and incubated in primary antibody to mitochondrial markers SOD2 (1:100, Assay Design) or complex V (1:500, Invitrogen Molecular Probes), 5mC (1:100, Calbiochem), Dnmt3a (1:100–500, Alexis or Abgent), or the autophagy marker LC3A (1:100, Cell Signaling). The sections were then rinsed, incubated in secondary antibody (1:400–800) conjugated to AlexaFluor-488 or -594, Hoechst dye counterstained (for nuclear visualization), and imaged on a Zeiss LSM 510 Meta confocal microscope or Zeiss Axiophot epifluorescence microscope.

### mtDNA isolation and mtDNA pyrosequencing

The PM fraction was used to isolate mtDNA using a Qiaprep Spin Miniprep kit (Qiagen) or conventional phenol-chloroform extraction, RNase digestion, and ethanol precipitation. mtDNA concentration and relative purity was determined by measuring A260 and A280 and calculating the A260/A280 ratio.

Mouse mtDNA was sequenced using Pyromark Q24 (Qiagen). DNA (2 μg) was bisulfite treated using an Epitek Bisulfite kit (Qiagen). Purified converted DNA (10 ng) was then PCR amplified, and 25 μl of product sequenced on the Pyromark Q24 (primers and conditions supplied by Qiagen using the Pyromark software): Locus 1 (D-loop), forward primer 5′- GGGTTTATTAAATTTGGGGGTAGTT-3′, biotinylated reverse primer 5′- ATAC CAAATA CATAACACCACAAT-3′, sequencing primer 5′- ATTTGGTTTTTACTTTAGGG T-3′; Locus 2 (16S rRNA gene), forward primer 5′- TGTTGGATTAGGATATTTTAATGGTGTAG-3′, biotinylated reverse primer 5′- CACCACCCTAATAACCTTCTCTA-3′, sequencing primer 5′- ATTTTAATGGTGTAGAAGT-3′; run conditions: 95°C 15 min, 45× (95°C 30 s, 58°C 30 s, 72°C 30 s), 72°C 5 min. The data was validated by internal controls and presented as percent 5mC/cytosine ± standard deviation (*n* = 3, in duplicate) with high agreement in duplicate measures.

### EM

Age-matched non-tg and hSOD1^mus^-G37R, -G93A, and -wildtype tg mice (15–17 months of age) received an anesthetic overdose and were perfused transcardially with 2% paraformaldehyde/2% glutaraldehyde. The group sizes were two mice per genotype. Tissue samples of left and right biceps femoris were acquired from each mouse and process and embedded in plastic for conventional transmission EM as described (Martin et al., 1994). Tissue samples were cut in the transverse plane at 0.5 μm for high-resolution light microscopy and then thin sections were cut and collected on copper grids for EM. These sections were viewed and imaged using a Phillips CM12 electron microscope. Digital electron micrographs from each mouse genotype were used to determine subsarcolemmal mitochondrial layer thickness and intermyofibrillar mitochondrial density in at least five images per mouse.

### Human CNS tissue

Rapid autopsy neurologic disease-free control human brain (*n* = 4) cerebral cortical samples were obtained through the Human Brain Resource Center at Johns Hopkins. These fresh samples of frontal cortex were used to isolate mitochondria for western blot studies of Dnmts.

### Statistical analysis

Western blot densitometry measurements, cytosine methylation pyrosequencing measurements, and myofiber mitochondrial density measurements were used to determine group means and variances and comparisons among groups were analyzed using a one-way analysis of variance and a Newman-Keuls *post-hoc* test or a Student's *t*-test.

## Results

### DNMT3A localizes to mitochondria

We analyzed several tissue types for the presence of Dnmts in mitochondria. A flow diagram for the mitochondrial purification preparation is shown (Figure 1, top), and the method was validated by western blotting (Figure 1, lower left). The fraction designated as PM was highly enriched for mitochondria, as determined by the enrichment of porin and cyclophilin D, and was essentially free of contamination from other subcellular organelles such as the endoplasmic reticulum (cytochrome p450), cytosol (actin), nucleus (lamin), and microsomes (catalase) (Figure 1, lower left). EM was used to show by direct visualization that the PM fraction had a high content of mitochondria (Figure 1, lower right).

We examined adult mouse skeletal muscle mitochondria for Dnmts (Figure 2A). The purity of the mitochondrial samples was confirmed by the enrichment of complex V (Figure 2A). Dnmt3a had a robust presence in skeletal muscle mitochondrial fractions (Figure 2A) and was detected with several different antibodies to Dnmt3a that recognize N-terminal, central, and C-terminal domains of the protein (Table 1). Dnmt1 was usually not detected in skeletal muscle PM fractions (Figure 2A), even after prolonged exposures and use of 3 different primary antibodies recognizing different domains of the protein (Table 1). In some mitochondrial preparations of skeletal muscle, Dnmt1 was detected at very low levels (Figure 2B) but was not present in fractions of intact mitochondria digested with agarose-bound proteinase K (Figures 2A,B). In contrast, Dnmt1 was highly concentrated in crude nucleus-enriched P1 fractions (Figure 1, Top) of the same tissue homogenates (Figures 2A,B). Dnmt3b was not detected in skeletal muscle mitochondria (data not shown).

To determine whether Dnmt3a is present within mitochondria or bound to the surface of mitochondria, fresh intact mitochondria were digested with agarose-bound proteinase K (Figure 2C). Tubulin, microtubule-associated proteins, and desmin are known to be tethered to the surface of striated muscle mitochondria (Capetanaki et al., 2007), and tubulin is known to specifically dock to VDAC (Carré et al., 2002). Proteinase K digestion removed completely or nearly completely mitochondrial-bound cytoskeletal proteins but did not alter the robust detection of Dnmt3a in PM (Figure 2C). This finding demonstrates that Dnmt3a is present within skeletal muscle mitochondria.

To confirm previous observations indicating a mitochondrial presence of Dnmt3a in nervous tissue (Chestnut et al., 2011), we tracked by subcellular fractionation the presence of Dnmt3a in mouse brain and spinal cord tissue (Figure 2D). Dnmt3a was present in nucleus-containing fractions (Figure 2D, lanes H and P1) and cytosol-containing fractions (Figure 2D, lanes H, S1, P2, and S2). Several isoforms of Dnmt3a have been reported (Chen et al., 2002).We detected both the 78 and 100 kDa isoforms of Dnmt3a in the different fractions of brain and spinal cord (Figure 2D). Brain and spinal cord PM fractions contained Dnmt3a (Figure 2D), as detected with three different antibodies to the N-terminal or central regions of Dnmt3a (Table 1). The 100 kDa isoform of Dnmt3a was concentrated in PM relative to total homogenate (Figure 2D). We were unable to detect Dnmt1 immunoreactivity in PM of brain and spinal cord using three different antibodies, consistent with previous data (Chestnut et al., 2011).

Western blotting for Dnmt3a in adult mouse skeletal muscle and spinal cord in a side-by-side comparison revealed tissue differences in isoform distribution (Figure 2E). In skeletal muscle, the 78 kDa isoform predominates over the 100 kDa isoform (Figure 2F), but in spinal cord the 100 kDa isoform predominates (Figure 2E) and accounts for nearly 100% of the total amount of this isoform in spinal cord mitochondria (Figure 2F).

We next tested the hypothesis that mitochondrial Dnmt3a is tissue specific. We examined by immunoblotting the presence of Dnmt3a in highly PM isolated from several adult mouse tissues, including spleen, liver, kidney, lung, heart, and testes (Figure 2G). The mitochondrial fractions of brain, spinal, heart, and testes contained the 100 kDa isoform of Dnmt3a (Figure 2G). Mitochondrial Dnmt3a protein levels were very low or undetectable in spleen, liver, kidney, and lung. The faintly detectable mitochondrial Dnmt3a in spleen, kidney, and lung appeared as the 78 kDa isoform (Figure 2G). To determine if the mitochondrial presence of Dnmt3a was mouse-specific, we prepared PM from human cerebral cortex (frontal cortex) and cultured human embryonic kidney (HEK) 293 cells (Figure 2G). Dnmt3a was detected robustly in mitochondria of the human brain but not in HEK293 cells (Figure 2G). Thus, the mitochondrial localization of Dnmt3a occurs primarily in the mitochondria of excitable tissues in human and mouse.

### Mitochondrial DNMT3A levels are reduced in skeletal muscle and spinal cord of tg mouse models of als

**Figure 3:**
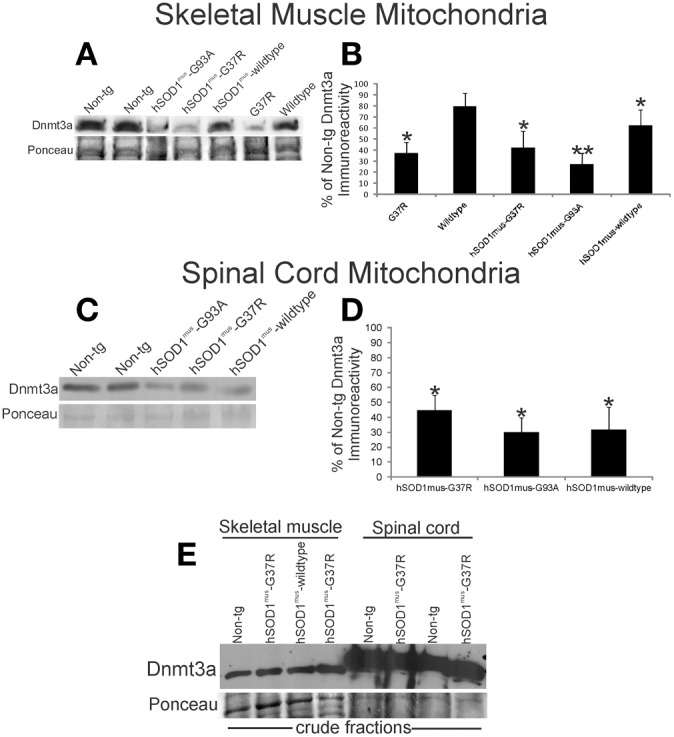
**Dnmt3a levels are reduced in hSOD1 tg mouse models of ALS**. **(A)** Western blot for Dnmt3a in leg skeletal muscle mitochondria (100 μg/lane) in non-conditional tg mice expressing hSOD1-G37R or hSOD1-wildtype constructs and conditional (muscle-restricted) tg mice expressing hSOD1^mus^-G37R, -G93A, or -wildtype constructs compared to age-matched non-tg littermates. Ponceau S staining of membrane shows protein loading. **(B)** Graph showing the relative levels of Dnmt3a immunoreactivity determined by immunoblotting of pure mitochondria isolated from hSOD1 tg mouse leg skeletal muscle. Values are mean ± SD. Significant differences from non-tg control are indicated by single (*p* < 0.05) or double (*p* < 0.01) asterisks. **(C)** Western blot for Dnmt3a in spinal cord mitochondria fractions (50 μg/lane) in conditional (muscle-restricted) tg mice expressing hSOD1^mus^-G37R, -G93A, or -wildtype constructs compared to age-matched non-tg littermates. Ponceau S staining of membrane shows protein loading. **(D)** Graph showing the relative levels of Dnmt3a immunoreactivity in hSOD1^mus^ tg mouse spinal cord. Values are mean ± SD. Significant differences from non-tg control are indicated by an asterisk (*p* < 0.05). The results have been replicated in at least 3 different experiments. **(E)** Western blot for Dnmt3a in crude fractions of skeletal muscle and spinal cord of conditional (muscle-restricted) tg mice expressing hSOD1^mus^-G37R or -wildtype constructs compared to age-matched non-tg littermates. Ponceau S staining of membrane shows protein loading.

Abnormalities in Dnmt protein levels and DNA methylation have been observed in human ALS CNS tissues and in mouse and cell models of motor neuron degeneration (Chestnut et al., 2011). Moreover, in human ALS skeletal muscle, marked mitochondrial abnormalities are found (Wiedemann et al., 1998; Vielhaber et al., 2000; Krasnianski et al., 2005; Echaniz-Laguna et al., 2006; Corti et al., 2009), and it has been proposed that skeletal muscle disease is a driving part of the disease process in ALS (Dupuis et al., 2006; Dobrowolny et al., 2008; Wong and Martin, 2010). We examined the levels of Dnmt3a in skeletal muscle mitochondria in hSOD1 tg mouse models of ALS at presymptomatic or early symptomatic stages of disease. Compared to non-tg age matched littermates, presymptomatic tg mice expressing mutant hSOD1-G37R non-conditionally in most tissues showed reduced levels of Dnmt3a in skeletal muscle mitochondria (Figures 3A,B). Tg early symptomatic mice with skeletal muscle-restricted expression of G37R mutant (hSOD1^mus^-G37R) and G93A mutant (hSOD1^mus^-G93A) also had a severe loss of skeletal muscle mitochondrial Dnmt3a (Figures 3A,B). Mitochondrial Dnmt3a protein levels were reduced modestly in hSOD1^mus^-wildtype tg mice but were not changed significantly in tg mice with non-conditional expression hSOD1-wildtype allele (Figures 3A,B). Spinal cords of hSOD1^mus^-G37R, -G93A, and -wildtype tg male mice at early stages of disease also had marked reduction in mitochondrial Dnmt3a (Figures 3C,D). The loss of Dnmt3a was selective for the mitochondrial compartment because Dnmt3a levels in crude homogenates of skeletal muscle and spinal cord were unchanged in tg mice (Figure 3E).

### Cellular localization of DNMT3A and 5mC in spinal cord and skeletal muscle and aberrant patterns of immunoreactivity in hSOD1^mus^ tg mice

**Figure 4:**
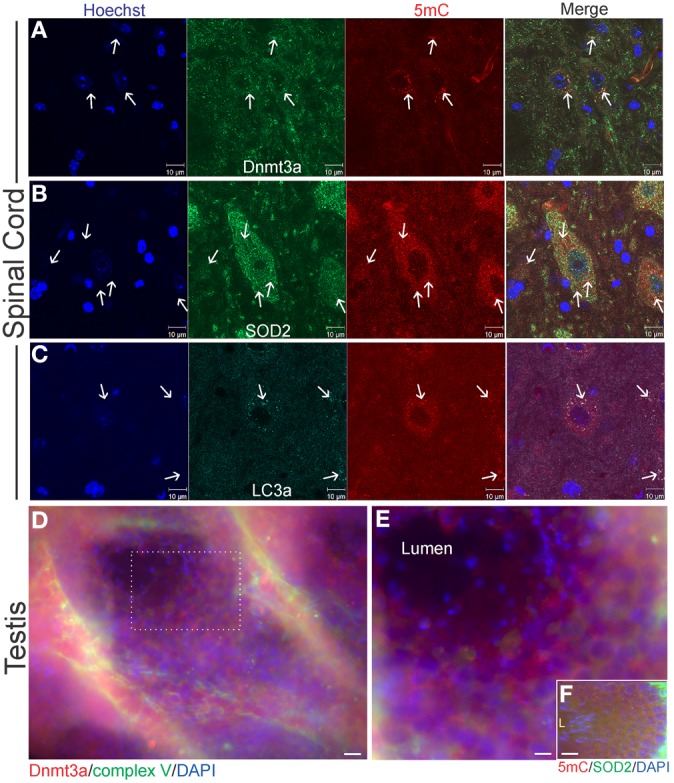
**Cellular localizations of Dnmt3a and 5-methylcytosine (5mC) in wildtype young adult mouse spinal cord and testes**. **(A)** In 6–8 weeks old non-tg mice, Dnmt3a is present in the nucleus and in the cytoplasm of neurons in spinal cord. Extranuclear cytoplasmic Dnmt3a immunoreactivity (green) is localized diffusely and in discrete bodies (arrows). These discrete bodies also contain 5mC (red, arrows). Hoechst staining was used to identify cell nuclei. **(B)** Cytoplasmic 5mC is localized to mitochondria in neurons. Some mitochondria, identified by SOD2 immunoreactivity (green, arrows), in spinal cord colocalize with 5mC immunoreactivity (red, arrows). Many mitochondria also do not contain 5mC. The SOD2- and 5mC-positive bodies tend to be larger than normal mitochondria (white arrows). **(C)** 5mC is present in autophagosomes. Mouse spinal cord sections were immunostained for an autophagosome marker LC3A (green) and 5mC (red). Extranuclear 5mC immunoreactivity has a high colocalization with LC3A in autophagosomes (arrows). **(D,E)** Immunofluorescent localization of Dnmt3a (red) and complex V (green) in mouse testis. Cell nuclei are blue. Dnmt3a immunoreactivity (red) is enriched in cells in the seminiferous tubules of testes. The boxed area in D is represented at higher magnification in E which shows that Dnmt3a immunoreactivity (red) partly colocalizes (seen as yellow-orange) with mitochondria (green). Scale bars: 30 μm **(D)**; 15 μm **(E)**. **(F)** Immunofluorescence for 5mC (red) and SOD2 (green) in mouse testes. Cell nuclei are blue. 5mC immunoreactivity (red) partly colocalizes (seen as yellow-orange) with mitochondria (green). Mature spermatozoa with scant mitochondria are seen at left in seminiferous tubule lumen (L). Scale bar = 30 μm.

Our biochemical finding that Dnmt3a is present in excitable tissue mitochondria required direct confirmation of a mitochondrial Dnmt3a localization *in situ* and an assessment of whether Dnmt3a in mitochondria overlaps with a reporter molecule for DNA methylation. We also assessed whether abnormalities in Dnmt3a and 5mC are found in tg mouse models of ALS since abrnomalities has been found in human ALS (Chestnut et al., 2011). We used a highly specific monoclonal antibody to detect 5mC (Kang et al., 2006). We have done additional characterization of the 5mC antibody (Chestnut et al., 2011). By immunofluorescence, we visualized Dnmt3a and 5mC localization within spinal cord of 2–4 months old non-tg mice (Figure 4A). Extranuclear Dnmt3a and 5mC immunoreactivities colocalized. While Dnmt3a immunoreactivity was present diffusely in the cytoplasm, there were distinct punctate structures positive for Dnmt3a. These cytoplasmic puncta invariably were 5mC-positive (Figure 4A, arrows), indicating that these structures contain methylated DNA. Consistent with expectations, 5mC immunoreactivity was also detected in the nucleus of neurons (Figures 4B,C, red).

As the only extranuclear cellular structures known to contain DNA are mitochondria, we examined spinal cord for the presence of 5mC within mitochondria. Using immunofluorescence for SOD2, a mitochondrial marker that shows robust punctate labeling in the cytoplasm (Martin et al., 2007), we determined that mitochondria contain 5mC (Figure 4B). Not all SOD2-positive structures were also positive for 5mC (Figure 4B, arrows). The dual positive structures often appeared larger than the SOD2-only positive mitochondria.

Some of the discreet cytoplasmic puncta that contained 5mC appeared similar to autophagosomes (Martin et al., 1994; Kabeya et al., 2000) or granules based on size and shape (Figure 4A). The larger 5mC-positive bodies were not positive for the mitochondrial marker SOD2, while the smaller 5mC-positive bodies did stain for SOD2 (Figure 4B), possibly indicating degradation of mitochondria or mitochondrial marker. We next examined the immunolocalization of 5mC and LC3A (Figure 4C). LC3A is a marker for autophagosomes (Kabeya et al., 2000) that is known to function in mitophagy (Wang and Klionsky, 2011). 5mC-containing, SOD2-negative bodies were positive for LC3A (Figure 4C). The prominent colocalization of 5mC and LC3A in the cytoplasm of spinal motor neurons suggests a relationship between cytosine methylation of mtDNA and mitophagy signaling.

In the mouse tissue screening for Dnmt3a protein levels we saw that testes also contained relatively high levels of Dnmt3a (Figure 2G). In seminiferous tubules, Dnmt3a and 5mC were discretely localized to mitochondria of spermatocytes (Figures 4D,E). Interestingly, immunofluorescent signal for mitochondria, Dnmt3a, and 5mC dissipated with maturation of spermatocytes to spermatozoa within the seminiferous tubule lumen (Figures 4D,E) consistent with a predominant pre-fertilization elimination of paternal mitochondria and mtDNA in sperm cells during their maturation, rather than a post-fertilization mechanism (Luo et al., 2013).

**Figure 5:**
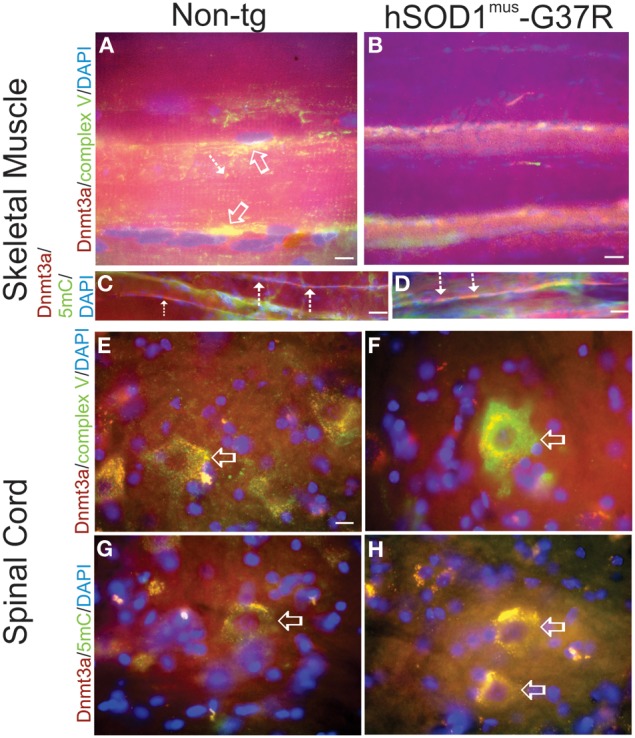
**Immunofluorescent localizations of Dnmt3a, mitochondria, and 5-methylcytosine (5mC) in hSOD1^mus^ tg mouse skeletal muscle and spinal cord**. **(A,B)** In non-tg mouse skeletal muscle (biceps femoris), Dnmt3a (red) immunoreactivity can be found diffusely in the sarcoplasm and associated with mitochondria (colocalization is seen as yellow) identified by complex V immunoreactivity (green). Dnmt3a/complex V colocalization occur prominently in subsarcolemmal mitochondria in non-tg mice (A, open arrows) and in interfibrillar mitochondria **(A**, hatched arrow**)**. In hSOD1^mus^ mouse hindleg skeletal muscle, Dnmt3a (red) immunoreactivity and mitochondrial complex V immunoreactivity (green) are markedly attenuated. Scale bars = 9 μm **(A)**, 15 μm **(B)**. **(C,D)**. Myofiber perinuclear colocalization of Dnmt3a and 5mC is intensified in hSOD1^mus^ tg mice. In non-tg mouse skeletal muscle (biceps femoris), Dnmt3a (red) immunoreactivity can be found clustered around peripheral myonuclei **(C**, hatched arrows**)** and generally has little colocalization with 5mC. In hSOD1^mus^ mouse skeletal muscle Dnmt3a (red) and 5mC (green) immunoreactivities have prominent perinuclear colocalizations **(D**, hatched arrows, yellow-orange**)**. Scale bars = 20 μm **(C,D)**. **(E,F)** In ~17 months old non-tg mice, Dnmt3a immunoreactivity (red) is present in the nucleus and, more prominently, in the cytoplasm of spinal cord motor neurons **(E,G**, open arrows**)**. Cytoplasmic Dnmt3a immunoreactivity (red) is localized in discreet particles in motor neurons **(E**,**G**, arrows**)**. Many of these cytoplasmic particles colocalize (seen as yellow) with complex V immunoreactivity **(E**, green**)**, and thus are mitochondria, and with 5mC **(G**, green, arrow**)** and thus contain methylated mtDNA. Scale bar in **(E) (**same for **F–H)** = 6 μm. In age-matched hSOD1^mus^ tg mice, remaining spinal motor neurons **(F**, open arrow**)** show intensified mitochondrial immunoreactivity for complex V (green) and the Dnmt3a immunoreactivity largely is colocalized with complex V **(F)** and 5mC **(H)** and is aggregated in the cytoplasm.

Immunofluorescent assessments of Dnmt3a and 5mC were done on skeletal muscle and spinal cord sections of hSOD1^mus^ tg mice to confirm biochemical findings and to put our homogenate-based observations into a cellular localization framework. In non-tg control mice, Dnmt3a colocalized with complex V robustly in subsarcolemmal mitochondria and also in intermyofibrillar mitochondria (Figure 5A), while Dnmt3a and complex V immunoreactivity was severely attenuated in hSOD1^mus^-G37R skeletal muscle myofibers (Figure 5B). In subsarcolemmal mitochondria adjacent to myonuclei, Dnmt3a was not colocalized with 5mC in non-tg controls (Figure 5C), but in hSOD1^mus^ tg mice, Dnmt3a and 5mC were prominently colocalized in apparent subsarcolemmal mitochondria (Figure 4D). 5mC immunoreativity was also seen in subsets of myofiber nuclei (Figures 5C,D, green). In lumbar spinal cord motor neurons of 15–17 months old non-tg mice, Dnmt3a was normally colocalized with complex V in about a third of the mitochondria in motor neuron cell bodies (Figure 5E) and, similarly, about a third of the Dnmt3-positive cytoplasmic particles colocalized with 5mC (Figure 5G). In hSOD1^mus^ tg mice, complex V immunoreactivity was enriched in remaining lumbar motor neurons and Dnmt3 immunoreactivity appeared as aggregates associated with some mitochondria (Figure 5F). The Dnmt3a-5mC colocalization in remaining mitochondria in motor neurons of hSOD1^mus^ tg mice also revealed large numerous cytoplasmic aggregates containing 5mC (Figure 5H) consistent with findings in degenerating motor neurons (Chestnut et al., 2011).

### Direct demonstration of 5mC in mtDNA

**Figure 6:**
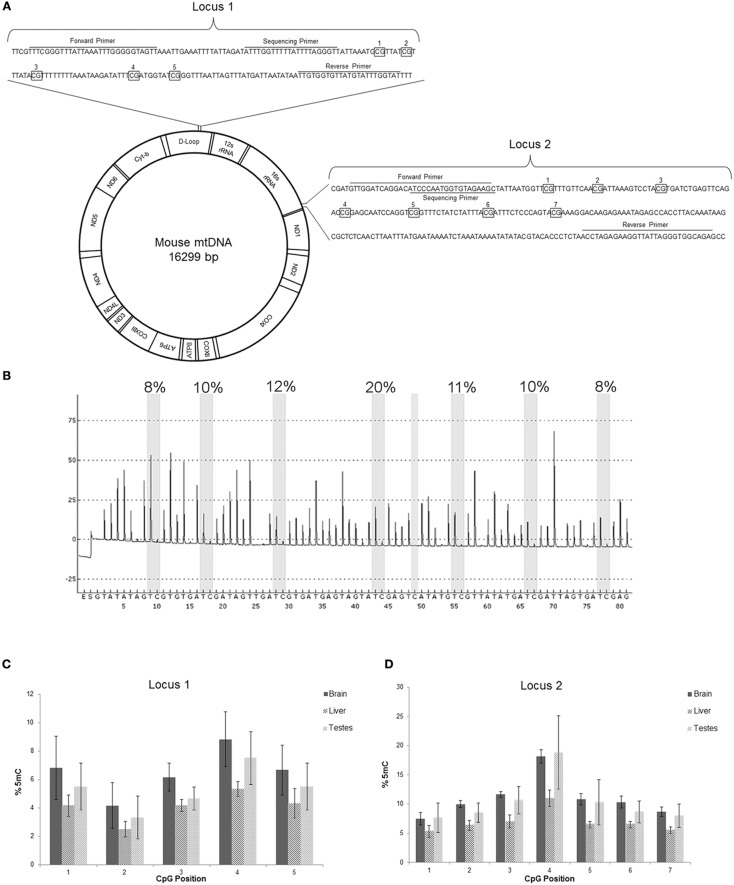
**mtDNA contains 5-methylcytosine (5mC)**. **(A)** Schematic of mouse mtDNA (Bibb et al., 1981) with depictions of the two locations assayed for 5mC. The first locus is in the D-loop, involved in mtDNA replication, and the second locus in the 16s rRNA encoding region. The primer locations are shown in sequences (wildtype or converted shown) with the CpG regions examined that are boxed and numbered. **(B)** Representative pyrogram from pyrosequencing of mouse brain mtDNA locus 2. The Y-axis is relative intensity (in arbitrary units) and the X-axis is the dispensation sequence showing the order of nucleotide addition to the reaction (E, enzyme; S, substrate). The seven thick shaded bars indicate the CpG positions with the degree of methylation (shown at top of bar) calculated from the ratio of the peak heights of C and T. The single narrow bar shows a bisulfite treatment control point where the peak only at the T dispensation and not at the C dispensation confirms full conversion by the bisulfite treatment. **(C,D)** Pyosequencing data of the two regions of mtDNA from mouse brain, liver, and testes. Numbers on the x-axis correspond to the CpG numbering in the sequence in **(A)**, and the y-axis is mtDNA 5mC content. In locus 1 the 5mC content was highest in brain mtDNA. In locus 2 the 5mC content in mtDNA was highest in the brain and testes, especially at position 4. Of the 3 tissues, liver had the lowest mtDNA 5mC content.

We directly identified 5mC in purified mtDNA by DNA pyrosequencing (Marsh, 2007) (Figures 6A,B). Two different loci of the mitochondrial genome (D-loop and 16S rRNA gene) were interrogated in bisulfite-treated mtDNA isolated from mouse brain, liver, and testes (Figure 6A). Cytosine methylation was determined at five nucleotide sites in locus 1 and at seven nucleotide sites in locus 2 (Figures 6A,B). Locus 1 in the D-loop had CpG regions with 5mC content ranging from 9% (brain, position 4) to 2% (liver, position 2) (Figure 6C). Most of the mtDNA CpG sites in locus 1 of brain and testes had 5mC content at ~4–6% (Figure 6C). Liver mtDNA was generally at 4% 5mC content at locus 1 (Figure 6C). In contrast, locus 2 in the 16S rRNA gene had several CpG regions with 5mC content ranging from 7.5 to 18% in brain and testes mtDNA and testes (Figure 6D). Locus 2 CpG position 4 showed the highest 5mC content (Figures 6B,D). Liver mtDNA generally had ~5% 5mC content at locus 2, with the exception of CpG position 4 where it was about 10% (Figure 6D).

### Als mice have aberrant mtDNA 5mC signatures

**Figure 7:**
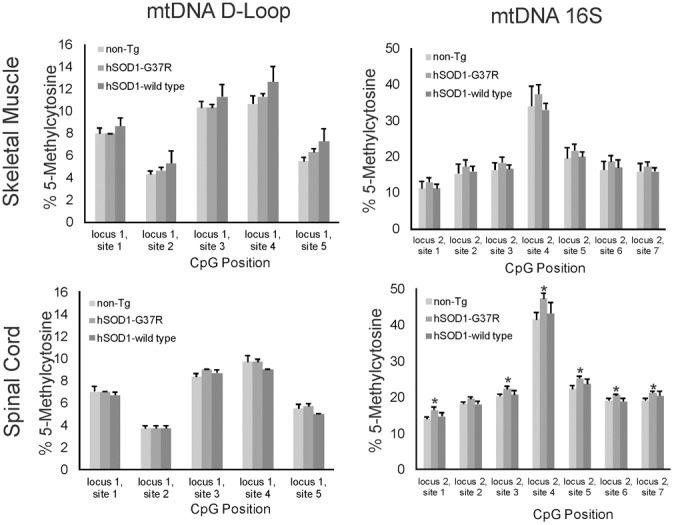
**5-methylcytosine (5mC) content in two loci of skeletal muscle mtDNA in non-conditional transgenic mouse models of ALS**. Graphs show the %5mC content at 5 CpG sites in the D-loop (**left**) and at 7 CpG sites in the 16S rRNA gene in the skeletal muscle (**top**) and spinal cord (**bottom**) of tg mice with expression of G37R or wildtype hSOD1 variants. Tg and non-tg control mice were 8–10 months of age. Values are mean ± SEM derived from mtDNA isolated from three different mice per genotype. Asterisks denote significant increase in hSOD1-G37R mice compared to non-tg control (*p* < 0.05).

**Figure 8:**
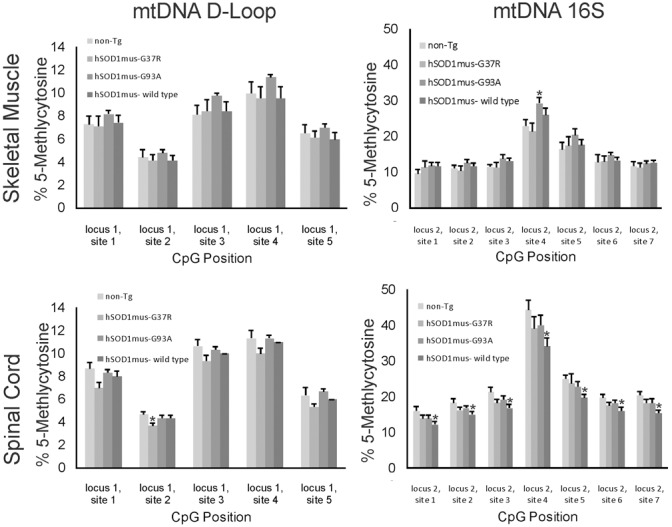
**5-methylcytosine (5mC) content in two loci of skeletal muscle mtDNA in conditional transgenic mouse models of ALS**. Graphs show the %5mC content at 5 CpG sites in the D-loop (**left**) and at 7 CpG sites in the16S rRNA gene in the skeletal muscle (**top**) and spinal cord (**bottom**) of tg mice with skeletal muscel-restricted expression of G37R, G93A, or wildtype hSOD1 variants. Tg and non-tg control mice were 16–20 months of age. Values are mean ± SEM derived from mtDNA isolated from three different mice per genotype. Asterisks denote significant differences in tg mice compared to non-tg control mice (*p* < 0.05).

Cytosine methylation in mtDNA was assayed in skeletal muscle and spinal cord of different hSOD1 tg mouse models of ALS (Figures 7, 8). Tg mice with non-conditional expression of mutant-G37R and wildtype variants of hSOD1 were analyzed at 8–10 months of age (Figure 7). Generally, 5mC content was lower in the D loop (locus 1) than in the 16S rRNA gene (locus 2) in control mice. Within the D-loop, the CpG at site 2 had the lowest % methylation, and sites 3 and 4 had the highest cytosine methylation in skeletal muscle and spinal cord (Figure 7). No significant differences in cytosine methylation of the D-loop were seen in tg mice expressing mutant-G37R and wildtype variants of hSOD1 compared to non-tg mice. In the 16S rRNA gene (locus 2), the CpG at site 4 had the highest % methylation of all the sites sequenced (Figure 7). Cytosine methylation was significantly increased at sites 1, 3, 4, 5, 6, and 7 in the 16S rRNA gene of spinal cord in tg mice with non-conditional expression of mutant-G37R (Figure 7), but no differences were detected in cytosine methylation in the 16S rRNA gene of skeletal muscle (Figure 7). Tg mice (16–20 months old) with skeletal muscle-restricted expression of mutant-G37R and -G93A and wildtype variants of hSOD1 (Figure 8) showed patterns of mtDNA cytosine methylation that were different compared to non-conditional hSOD1 tg mice. Cytosine methylation in D-loop site 2 of spinal cord was significantly reduced in hSOD1^mus^-G37R (Figure 8), but other sites assayed for in the D-loop of spinal cord and skeletal muscle were unchanged (Figure 8). In contrast, cytosine methylation in all sites of the 16S rRNA gene of spinal cord was significantly lower in hSOD1^mus^-wildtype mice compared to non-tg mice (Figure 8), but G37R^mus^ and G93A^mus^ mice did not differ from control.

### hSOD1^mus^ tg mice develop mitochondrial abnormalities in skeletal muscle and spinal cord

**Figure 9:**
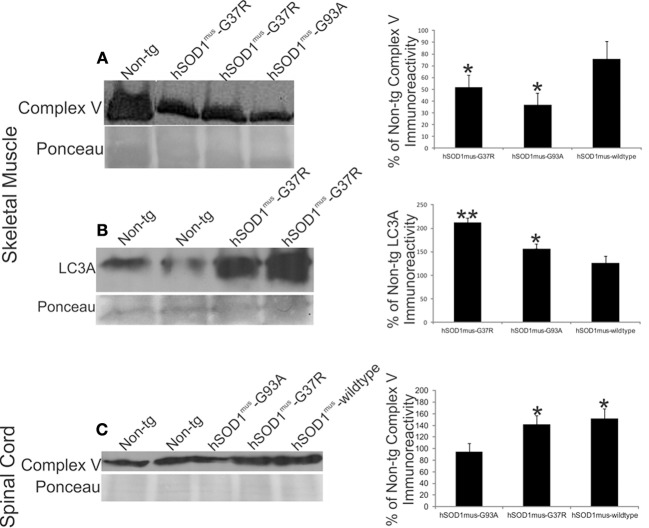
**hSOD1^mus^ tg mouse model of ALS has mitochondrial abnormalities in skeletal muscle and spinal cord**. **(A)** Western blot for complex V in skeletal muscle crude extracts (100 μg/lane) of early symptomatic tg mice expressing skeletal muscle-restricted hSOD1^mus^-G37R or hSOD1^mus^-wildtype. Ponceau S-stained membrane shows protein loading. Graph (at right) shows the quantification of complex V immunoreactivity in early symptomatic hSOD1^mus^-G37R, hSOD1^mus^-G93A, or hSOD1^mus^-wildtype tg mice. Values (as % of control) are mean ± SD. Asterisk denotes significant differences (*p* < 0.01) from non-tg control. **(B)** Western blot for autophagy marker LC3A in skeletal muscle crude extracts (100 μg/lane) early symptomatic tg mice expressing skeletal muscle-restricted hSOD1^mus^-G37R or hSOD1^mus^-wildtype. Ponceau S-stained membrane shows protein loading. Graph (at right) shows the quantification of LC3A immunoreactivity in early symptomatic hSOD1^mus^-G37R, hSOD1^mus^-G93A, or hSOD1^mus^-wildtype tg mice. Values (as % of control) are mean ± SD. Significant differences from non-tg control are indicated by single (*p* < 0.05) or double (*p* < 0.01) asterisks. **(C)** Western blot for complex V in spinal cord crude extracts (50 μg/lane) of early symptomatic tg mice expressing skeletal muscle-restricted hSOD1^mus^-G37R or hSOD1^mus^-wildtype. Ponceau S-stained membrane shows protein loading. Graph (at right) shows the quantification of complex V immunoreactivity in spinal cord of early symptomatic hSOD1^mus^-G37R, hSOD1^mus^-G93A, or hSOD1^mus^-wildtype tg mice. Values (as % of control) are mean ± SD. Asterisk denotes significant differences (*p* < 0.05) from non-tg control.

The loss of mitochondrial Dnmt3a seen in tg mouse models of ALS could be related to changes in mitochondrial content. Indeed, skeletal muscle complex V was reduced significantly in hSOD1^mus^-G37R, -G93A, and -wildtype tg mice at middle stages of disease compared to age-matched non-tg mice (Figure 9A). The reduced levels of complex V in skeletal muscle were mirrored by increases in the autophagy marker LC3A (Figure 9B). In contrast, complex V levels in spinal cord were significantly elevated in hSOD1^mus^ tg mice (Figure 9C), consistent with previous histologic observations showing increased numbers of mitochondria in motor neurons of these mice (Wong and Martin, 2010).

**Figure 10:**
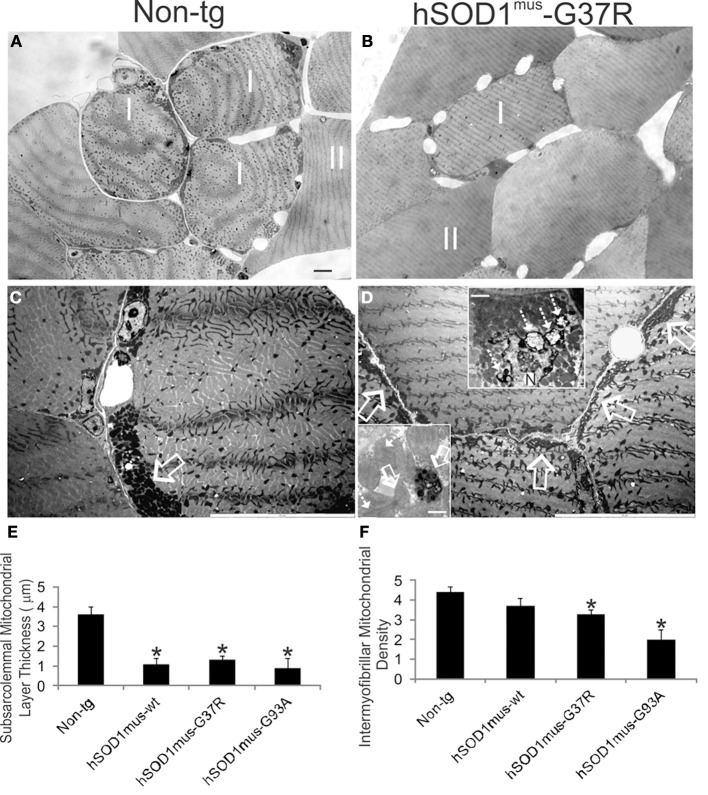
**hSOD1^mus^ tg mouse model of ALS shows loss of mitochondria in skeletal muscle**. **(A,B)** Toluidine blue-stained plastic sections (0.5 μm thick) of biceps femoris muscle from 15 month-old non-tg and hSOD1^mus^-G37R tg mice. In non-tg mice, type I and type II fibers have distinct appearances as evidenced by the surrounding capillary number and subsarcolemmal and interfibrillar mitochondria density. In tg mice, type I fibers are less prominent with apparent loss of subsarcolemmal and interfibrillar mitochondria. Scale bar (in **A**, same for **B**) = 8 μm. **(C,D)** Electron micrographs showing a high density of subsarcolemmal mitochondria in non-tg mice **(C**, open arrows**)** and prominent attenuation of the subsarcolemmal layer in hSOD1^mus^ tg mice **(D**, open arrows**)**. Upper inset **(D)** shows prominent mitophagy of subsarcolemmal mitochondria (hatched arrows) by a myonucleus (N) in hSOD1^mus^-G37R tg mouse skeletal muscle. Scale bar = 1.3 μm. Lower inset **(D)** shows degenerating mitochondria with round inclusions (open white arrows) or rod inclusions (hatched white arrows) from hSOD1^mus^-G93A tg mouse skeletal muscle. Scale bar = 0.5 μm. **(E)** Graph of the thickness of type I fiber subsarcolemmal mitochondrial layers in age-matched (~17 months) non-tg and hSOD1^mus^ tg mice. Values are mean ± SD. Asterisk denotes significant differences (*p* < 0.01) from non-tg control. **(F)** Graph of type II fiber interfibrillar mitochondrial densities in age-matched (~17 months) non-tg and hSOD1^mus^ tg mice. Values are mean ± SD. Asterisk denotes significant differences (*p* < 0.01) from non-tg control.

Mitochondrial distributions and numbers in skeletal muscle were examined using high-resolution light and transmission electron microscopic evaluation of plastic sections. Non-tg control mice were rich in subsarcolemmal and interfibrillar mitochondria (Figures 10A,C). hSOD1^mus^ tg mice showed a loss of subsarcolemmal and interfibrillar mitochondria (Figures 10B,D,E). In putative type I fibers of non-tg mice the subsarcolemmal mitochondrial layer thickness was ~4 μm (Figures 10C,E), but in hSOD1^mus^-G37R, -G93A, and -wildtype tg mice, the subsarcolemmal mitochondrial layer was significantly attenuated (Figure 10E). Putative type II fibers in hSOD1^mus^ tg mouse skeletal muscle displayed a significantly reduced density of interfibrillar mitochondria (Figure 10F) compared to age-matched non-tg mice. EM additionally revealed evidence for subsarcolemmal and interfibrillar mitophagy (Figure 10D, upper inset), as well as intramitochondrial rod-like inclusions (Figure 10D, lower inset) indicative of mitochondrial myopathy (Schlattner et al., 2006).

## Discussion

We demonstrate that the DNA methylating enzyme Dnmt3a is present in mitochondria of mouse CNS, striated muscle, and testes. We also find Dnmt3a in human cerebral cortex mitochondria. Furthermore, the mtDNA of these tissues contains 5mC as shown directly by DNA pyrosequencing. Dnmt3a protein levels are reduced significantly at early disease in skeletal muscle and spinal cord of tg mouse models of ALS, and these mice also show aberrant patterns in 5mC immunoreactivity in skeletal muscle and spinal motor neurons. Some 5mC-positive structures are sequestered into autophagosomes. The skeletal muscle of presymptomatic tg mouse models of ALS shows accumulation of autophagy marker, loss of subsarcolemmal and intermyofibrillar mitochondria, and ultrastructural evidence for mitophagy. We conclude that mitochondrial localization of Dnmt3a and cytosine methylation of mtDNA are tissue-preferential, mostly confined to excitable tissues, and that regulatory mechanisms for epigenetic modification mtDNA, or non-catalytic functions of mitochondrial Dnmt3a, in skeletal muscle and spinal cord are aberrant in mouse ALS.

DNA methylating enzymes are thought traditionally to localize and function in the cell nucleus where they catalyze cytosine methylation leading to chromatin remodeling (Cheng, 1995; Jones and Takai, 2001; Bird, 2002; Brenner and Fuks, 2006). Here, we show that Dnmt3a is present the mitochondria of adult mouse excitable tissues such as brain, spinal cord, heart, and skeletal muscle. Mitochondrial Dnmt3a was also present in testes. In contrast, Dnmt3a is low or undetectable in spleen, liver, kidney, and lung. We detected mitochondrial Dnmt3a with several different antibodies to Dnmt3a that recognize different epitopes. The finding was not an artifact of subcellular contamination because the mitochondrial preparation was assessed for purity by immunoblotting and EM. Moreover, the Dnmt3a was present within mitochondria rather than being docked to the surface of mitochondria, as was the case for the mitochondrial association of Dnmt1. We also observed that different Dnmt3a isoforms were enriched preferentially in different mouse tissues. Skeletal muscle expressed primarily the 78 kDa Dnmt3a isoform, whereas, nervous tissue expressed primarily the 100 kDa Dnmt3a isoform. Dnmt3a was found also in mitochondria purified from adult human cerebral cortex. Dnmt3b was not detected in mouse skeletal muscle mitochondria (data not shown). We confirmed the mitochondrial expression of Dnmt3a by immunolocalization. Dnmt3a was found to colocalize with mitochondrial markers in spinal cord, skeletal muscle and testes. Another group has shown the presence of a Dnmt isoform associated with mitochondria, but the mitochondria were prepared from cell cultures and only one antibody to Dnmt1 and one antibody putatively to Dnmt3a was used (Shock et al., 2011). Dnmt1, but not Dnmt3a, was found associated with mitochondria from cultured mouse embryonic fibroblasts and human colon carcinoma cells (Shock et al., 2011), but mitochondrial surface-associated Dnmt1 was not ruled out by protease digestion of outer membrane-bound proteins as done here. We did not detect Dnmt1 in mouse skeletal muscle mitochondria using an antibody specifically directed to the N-terminus of Dnmt1. A commercial antibody (Abcam) to amino acids 1–10 of the N-terminus of Dnmt1 was reportedly used in this previous study (Shock et al., 2011), but this antibody was not in inventory for our use. With antibodies to N-terminal and central regions of Dnmt1, we identified here a proteinase K-sensitive Dnmt1 in some mitochondrial preparations, indicating surface-associated Dnmt1. Dnmt1 is associated with imprinting mechanisms involved in maintaining methylation patterns during cell division (Robertson, 2001), likely to be occurring in cultured cycling cells, while Dnmt3a has *de novo* methylating activity (Okano et al., 1999). We conclude that the major mitochondrial Dnmt in adult mouse and human excitable tissues *in vivo* is Dnmt3a.

Our finding that Dnmt3a is within mitochondria of excitable tissue led to measurements of mtDNA methylation in these tissues. We demonstrated directly the presence of 5mC in mtDNA by DNA pyrosequencing (Marsh, 2007). Because DNA pyrosequencing cannot distinguish between 5mC and 5-hydroxymethylcytosine, our pryosequencing data represents a measurement of 5mC and 5-hydroxymethylcytosine in mtDNA. A recent study has also demonstrated the presence of 5mC and 5-hydroxymethylcytosine in mtDNA of cultured mouse embryonic fibroblasts and human colon carcinoma cells using qPCR and Gla1 restriction enzyme digestion (Shock et al., 2011). While it is possible the mtDNA methylation is involved in regulating gene expression (Shock et al., 2011), as in the nucleus, the proteins involved in chromatin remodeling, such as histones, are not present in mitochondria (Spelbrink, 2010). This suggests the intriguing possibility that cytosine methylation through Dnmt3a is a major epigenetic mechanism involved in mitochondrial gene regulation without partnership with histone acetylation. Alternatively, the mtDNA methylation may serve some purpose other than gene regulation.

Our immunolocalization experiments on mouse skeletal muscle, spinal cord, and testes revealed that some 5mC-positive structures in the cytoplasm of myofibers, spinal motor neurons, and maturing spermatozoa were mitochondria because the 5mC colocalized with SOD2 or complex V and Dnmt3a. This observation confirms a previous report of mitochondrial 5mC localization using other mitochondrial makers (Chestnut et al., 2011). However, some cytoplasmic 5mC-positive structures were not positive for mitochondrial markers and had a morphology different from mitochondria because they were larger than typical mitochondria and sometimes appeared aggregated. We thus sought to determine possible relationships between methylated mtDNA and mitophagy and found that 5mC colocalized with the autophagosome marker LC3A (Klionsky and Emr, 2000). Our findings in mouse testes suggest that enhanced mtDNA methylation and mitophagy in maturing spermatoza in seminiferous tubules could be a mechanism to reduce the major burden of paternal mtDNA transmission prior to postfertilization autophagy (Al Rawi et al., 2011). Autophagy has also been associated with nervous system and muscle disease (Batlevi and La Spada, 2011). In hSOD1 tg mice with muscle-specific expression, large 5mC-positive aggregates were observed in the cytoplasm of subsets of spinal motor neurons. Similar observations have been shown in adult mouse spinal motor neurons undergoing axotomy-induced apoptosis (Chestnut et al., 2011). The presence of Dnmt3a and 5mC within a subset of mitochondria, many of which are associated with autophagosomes, suggests an upstream epigenetic mechanism for mitophagy in the regulation of normal and pathological mitochondrial dynamics.

We have found that epigenetic mechanisms involving DNA methylation can drive motor neuron apoptosis in cell culture and *in vivo* and that aberrant regulation of DNA methylation is part of the pathobiology of human ALS (Chestnut et al., 2011). Abnormalities in the levels and localizations of Dnmt1, Dnmt3a, and 5mC have been found in human ALS spinal cord and motor cortex (Chestnut et al., 2011), and mitochondrial abnormalities in skeletal muscle, liver, spinal motor neurons, and motor cortex have been reported in human ALS (Sasaki and Iwata, 1999; Menzies et al., 2002). However, causal disease mechanisms are difficult to pinpoint using human postmortem tissue. We therefore evaluated Dnmt3a and mtDNA methylation in several tg mouse models of ALS expressing mutant and wildtype hSOD1 either non-conditionally with global tissue expression (Gurney et al., 1994; Martin et al., 2007, 2009; Gertz et al., 2012) or conditionally with muscle-restricted expression (Wong and Martin, 2010). In skeletal muscle, mitochondrial Dnmt3a levels were markedly reduced in most hSOD1 tg mouse lines at stages of disease ranging from presymptomatic to mid-symptomatic. The loss of Dnmt3a and mitochondria in skeletal muscle was corroborated microscopically by immunohistochemistry. Interestingly, spinal cord Dnmt3a was reduced in tg mice with muscle-specific expression of hSOD1. This change is interesting because conditional ablation of Dnmt3a specifically in nervous tissue causes an age-related ALS-like phenotype in mice involving neuromuscular junction dismantling, motor neuron loss, and shortened lifespan (Nguyen et al., 2007). The localizations of 5mC immunoreactivity in skeletal muscle and spinal cord were also different in non-conditional and muscle-specific hSOD1 tg mice compared to age-matched control mice. Skeletal muscle disease in ALS mice thus appears to trigger retrograde transynaptic changes in epigenetic DNA methylation machinery in spinal cord mitochondria.

Abnormalities in mtDNA cytosine methylation were detected by pyrosequencing in mouse models of ALS. The cytosines interrogated were in the D-loop and 16S ribosomal RNA gene (Figure 6A). The 16S ribosomal RNA gene was more vulnerable than the D-loop to anomalous cytosine methylation. In the 16S ribosomal RNA gene, six cytosine sites showed increased methylation in spinal cord of hSOD1-G37R mice, and one cytosine site showed increased methylation in skeletal muscle of hSOD1^mus^-G93A mice. While five cytosines in the D-loop showed differential levels of methylation, their methylation was similar in control and ALS mice. While the D-loop is generally thought to be a major regulator of mitochondrial genome transcription, it is now known that non-promoter CpG methylation within gene bodies can regulate gene expression in the nuclear genome (Maunakea et al., 2010). We have no direct explanation for the apparent mismatches between mitochondrial Dnmt3a protein levels which decrease and 5mC which increase. Recent studies have found that Dnmt3a has demethylase activity that is stimulated by Ca^2+^ ions (Chen et al., 2013), so the increased cytosine methylation could be construed as being consistent with the loss of Dnmt3a in mitochondria.

A mechanism for the loss of skeletal muscle mitochondria appears to be mitophagy, as evidenced by the ultrastructure of degenerating mitochondrial profiles and the marked upregulation of LC3A seen by western blotting. Because numerous copies of mtDNA reside in single mitochondrion, Dnmt3a protein levels may be more reflective of skeletal muscle mitochondrial numbers, and extensively methylated mtDNA may be more stable than Dnmt3a protein in mitochondria undergoing mitophagy. Recent evidence supports this possibility because some mtDNA can escape autophagy and participate in local inflammatory mechanisms (Oka et al., 2012). Tissue inflammation is a prominent component of the pathobiology of mouse models of ALS (Martin et al., 2007; Chen et al., 2010; Drechsel et al., 2012) and human ALS (McGeer and McGeer, 2002; Corcia et al., 2013).

## Author contributions

Conceived and designed the experiments: Margaret Wong, Barry Gertz, Barry A. Chestnut, Lee J. Martin. Performed experiments: Margaret Wong, Barry Gertz, Lee J. Martin. Analyzed the data: Margaret Wong, Barry Gertz, Lee J. Martin. Wrote paper: Margaret Wong, Barry Gertz, Lee J. Martin.

### Conflict of interest statement

The authors declare that the research was conducted in the absence of any commercial or financial relationships that could be construed as a potential conflict of interest.
